# Anxiety and depression amongst patients enrolled in a public sector antiretroviral treatment programme in South Africa: a cross-sectional study

**DOI:** 10.1186/1471-2458-12-244

**Published:** 2012-03-27

**Authors:** Michele Pappin, Edwin Wouters, Frederik LR Booysen

**Affiliations:** 1 Centre for Development Support, University of the Free State, Bloemfontein, South Africa; 2 Research Centre for Longitudinal and Life Course Studies (CELLO), University of Antwerp, Antwerp, Belgium; 3 Department of Economics, University of the Free State, Bloemfontein, South Africa

## Abstract

**Background:**

HIV/AIDS and depression are projected to be the two leading causes of disability by 2030. HIV/AIDS and anxiety/depression are interlinked. People suffering from depression may be more likely to engage in risky sexual behaviour, and therefore at greater risk of contracting HIV. An HIV + diagnosis may trigger symptoms of anxiety and depression, which may in turn result in risky sexual behaviour and the spread of HIV. This study explores correlates of anxiety and depression in patients enrolled in a public sector ART programme in South Africa.

**Methods:**

Interviews were conducted with 716 patients initiating ART at twelve public health care facilities in the Free State. Symptoms of anxiety and depression were measured using the Hospital Anxiety and Depression Scale (HADS). An 8+ cut-off was used to identify possible cases of anxiety and depression. Multivariate logistic regression analysis, using STATA Version 11, was performed to identify correlates of anxiety and depression.

**Results:**

The prevalence of symptoms of respectively anxiety and depression amongst this study population in the Free State was 30.6% and 25.4%. The multivariate logistic regression analyses identified five correlates of symptoms of anxiety and depression. Disruptive side effects (OR = 3.62, CI 1.95-6.74) and avoidant coping (OR = 1.42, CI 1.22-1.65) were associated with a greater number of symptoms of anxiety. Stigma was associated with an increase in symptoms of anxiety (OR = 1.14, CI 1.07-1.21) and of depression (OR = 1.13, CI 1.06-1.20), while being a widow (OR = 0.30, CI 0.13-0.69) and participating in a support group (OR = 0.21, CI 0.05-0.99) were associated with decreased symptoms of depression.

**Conclusions:**

The findings from the study provide valuable insights into the psychosocial aspects of the Free State public-sector ART programme. Combined with the literature on the intricate link between mental health problems and treatment outcomes our results emphasise firstly, the necessity that resources be allocated for both screening and treating mental health problems and, secondly, the need for interventions that will encourage support-group participation, address ART side effects, reduce maladaptive coping styles, and minimise the stigma associated with symptoms of anxiety and/or depression.

## Background

HIV/AIDS and depression are projected to be the world's two leading causes of disability by 2030 [1]. Worldwide, 33 million people are currently living with HIV. In 2009, there were an estimated 2.6 million new HIV infections and 1.8 million deaths due to AIDS [2]. Depression, on the other hand, affects 121 million people globally [3].

Importantly, HIV/AIDS and anxiety/depression are interlinked. People suffering from depression may be more likely to engage in risky sexual behaviour, and they are therefore at greater risk of contracting HIV [4-6]. Conversely, an HIV + diagnosis may trigger symptoms of anxiety and depression [7,8], which could once again lead to risky sexual behaviour and the spreading of the virus. In addition, studies have shown that people suffering from depression are less likely to adhere to treatment - treatment for both mental illness and for antiretroviral treatment (ART) [9]. Depression may therefore lead to non-adherence to ART and result in poorer health. Unfortunately, more than half of the HIV + population that suffer from depression have not received an official diagnosis of their depression [10].

Recent studies on the prevalence of anxiety and depression in HIV/AIDS patients who are on ART in resource-limited settings confirm this link. Symptoms of anxiety and depression among 386 people initiating ART in Brazil were measured using the Hospital, Anxiety and Depression Scale (HADS) (≤ 10). The prevalence of anxiety and depression, respectively, was 35.8% and 21.8% [11]. A study in Uganda assessed the prevalence of symptoms of depression in 1017 patients eligible for ART using the Centre for Epidemiologic Studies Depression Scale (≥ 23). The prevalence of depression was 47% [12].

A study in Cape Town, South Africa, assessed the prevalence of psychiatric disorders using the Mini International Neuropsychiatric Interview among 149 recently diagnosed HIV/AIDS patients. The prevalence of anxiety was 6.7% and that of depression was 34.9% [13,14], which highlights the extent of anxiety and/or depression in HIV + populations. Prevalence rates must however be interpreted with caution as location of study, study population and different types of measures make it impossible to arrive at a single prevalence rate for anxiety or depression. In addition, self-reported measures and psychiatric screening tools- such as HADS- report higher rates of depression than do diagnostic evaluations [15].

Researchers have identified a broad spectrum of protective and risk factors associated with anxiety and depression in HIV + populations. Apart from sociodemographic characteristics, such as age and gender, correlates include socio-economic (education, income, poverty), behavioural (alcohol use, sexual activity), psychosocial (disclosure, family support, prior depression, family history of depression, positive coping, stigma, and social support) and health-related factors (pain, health status, time on treatment) [11,13,16-28].

The complex interrelationship between these protective and risk factors correlated with mental health, the occurrence of anxiety and depression, and the health outcomes of ART recipients all render this issue an urgent research priority. However, previous research does not - in one and the same study - simultaneously incorporate all correlates of anxiety and depression in people receiving ART, despite the fact that such a comprehensive framework and knowledge is vital with a view to developing appropriate interventions. This study fills this particular research gap by simultaneously assessing the impact of demographic, socio-economic, behavioural, psychosocial, and health variables on symptoms of anxiety and depression in a population of public sector ART patients in the Free State Province of South Africa.

## Methods

### Data

This study is part of a cohort study entitled "Effective Aids Treatment and Support in the Free State (FEATS)" that was conducted by the Centre for Health Systems Research and Development (CHSR&D) of the University of the Free State (UFS). The study was approved by the Ethics Committee of the Faculty of Health Sciences of the UFS [ETOVS 145/07, DOH-27-0907-2025]. Study participants were recruited in 2007/08 from twelve public ART clinics in five districts in the Free State. Inclusion criteria were being eighteen years or older, having commenced ART in the five weeks prior to recruitment, and, residing in the town or village where the particular health facility was located. Data were collected by trained enumerators using structured face-to-face interviews once written informed consent had been obtained from study participants. The target sample size was 648 study participants and households. Because not all households consented to participate in the study, additional patients were recruited, this resulting in a total of 716 study participants.

### Measures

As specified in detail in the Appendix below, data on demographic, socio-economic, behavioural, psychosocial and health variables were collected. *Socio-economic *variables included: 'education level', 'dwelling type ',' financial statuses, and 'food supplements'. *Behavioural variables *comprised: 'use of alcohol', 'use of tobacco', 'use of dagga', and 'engagement in sexual activity'. *Psychosocial factors *included: 'stigma', 'coping', and 'psychosocial support'. Psychosocial support included the variables 'treatment buddy', 'community worker', 'support group' and 'disclosure' [29,30]*. Health-related variables *included 'health-related quality of life' (EQ-VAS) [31,32], 'time since first HIV + test', and 'ART side effects'.

In this study, anxiety and depression referred to self-reported symptoms of anxiety and depression and not Axis I anxiety and depressive clinical disorders. Symptoms of anxiety and depression were measured using HADS, which was originally developed as a self-administered tool to assess anxiety and depression in medically ill patients [33]. The scale contains four response options and comprises two subscales of seven questions each. The subscales measure anxiety and depression respectively and range from 0-21, with a higher score denoting a greater number of symptoms of either anxiety or depression. Previous psychometric investigations have shown that HADS achieves both good internal consistency and test-retest reliabilities, is sensitive to change, and provides valid assessments in HIV-positive populations [34,35] in resource-limited settings [36,37]. For the purposes of this study, the instrument was translated into SeSotho. The Cronbach's alphas for the subscales were 0.66 for anxiety and 0.61 for depression [38]. In this study, HADS scores were converted into a binary categorical variable, with an 8 + score being used to identify possible cases of clinical anxiety and depression [33,34]. A study in Brazil of HIV + women also used a score of greater than 7 to identify symptoms of anxiety or depression [36,37]. A review of the psychometric properties of HADS moreover found that "[O]ptimal balance between sensitivity and specificity for HADS as a screening instrument was achieved most frequently at a cut-off score of 8+ for both HADS-A[nxiety] and HADS-D[epression]" [39].

### Data analysis

STATA Version 11 was used to perform the statistical data analysis. Firstly, descriptive statistics of the anxiety and depression measures were generated with the aim of assessing the prevalence of anxiety and depression in the study population. Secondly, descriptive statistics of sociodemographic and ART characteristics of study participants were compiled. Finally, two sets of multivariate logistic regression analyses were run, using anxiety and depression as separate dependent variables to investigate correlates of anxiety and depressive symptoms independently. A literature review on HIV/AIDS, antiretroviral treatment, and anxiety and depression informed the specification of the multivariate logistic regression models [11,25,26,40,41]. The independent variables in the multivariate regression models were: residential district, gender, age, marital status, education level, dwelling type, treatment buddy, community worker, support group, positive coping, avoidant coping, seeking-social-support coping, stigma, disclosure, use of alcohol, use of tobacco, use of dagga, sexual activity, financial status, food supplements, time since first HIV test, ART side effects and health-related quality of life (EQ-VAS).

## Results

The majority of the study participants were black (98.4%) and female (75.7%). The mean age of the study participants was 37 years, and the median age was 36 years [IQR: 30.79-42.79]. Participants were mostly single (41.5%), while 23.7% were living with a spouse or partner, 14.6% were widowed and 10.6% were separated or divorced. The majority of the study participants lived in formal dwellings (66.6%) and 19.5% lived in informal dwellings. Most study participants had some education, mainly some secondary (45.8%) or primary education (31.0%). All the study participants had commenced ART, and the mean treatment duration was 37.7 days, with a median of 30 days [IQR: 18.0-46.5]. The prevalence of symptoms of anxiety was 30.6% and that for depression was 25.4%.

### Correlates of anxiety

The multivariate logistic regression analysis identified the following variables as correlates of symptoms of anxiety in people receiving ART (Table 1). HIV + patients who experienced very disruptive side effects from ART reported more of the symptoms of anxiety than did patients who had not reported side effects (OR = 3.62, CI 1.95-6.74). An avoidant coping style (OR = 1.42, CI 1.22-1.65) and stigma (OR = 1.14, CI 1.07-1.21) were also positively correlated with symptoms of anxiety. Finally, the longer study participants had known their HIV status, the more symptoms of anxiety they were likely to have experienced (OR = 1.01, CI 1.00-1.02).

**Table 1:** Multivariate logistic regression analysis of factors correlated with symptoms of anxiety in HIV + respondents on ARV treatment

	Odds Ratio	P-value	95% conf. Interval
District (comparison group = Lejweleputswa)			

Motheo	1.29		0.59 - 2.80

Fezile Dabi	1.06		0.51 - 2.20

Thabo Mofutsanyane	0.89		0.47 - 1.71

Xhariep	3.98		0.83 - 19.16

Female	0.70		0.38 - 1.28

Age (comparison group = under 30)			

30-34 years	0.79		0.35 - 1.80

35-39 years	0.52		0.23 - 1.16

40-44 years	1.13		0.50 - 2.54

45+ years	0.72		0.30 - 1.72

Marital status (comparison group = single)			

Cohabiting	0.55		0.27 - 1.11

Not cohabiting	0.72		0.34 - 1.56

Widowed	0.65		0.30 - 1.41

Separated or divorced	0.76		0.31 - 1.85

Education (comparison group = none)			

Primary	0.65		0.20 - 2.15

Some secondary	0.38		0.11 - 1.27

Matric and post-matric	0.49		0.14 - 1.78

Dwelling (comparison group = formal dwelling)			

Informal	0.92		0.48 - 1.74

Traditional	0.69		0.27 - 1.72

Other	0.41		0.17 - 1.00

Treatment buddy (yes/no)	0.91		0.67 - 1.25

Community worker (yes/no)	0.79		0.29 - 2.19

Support group (yes/no)	0.79		0.20 - 3.18

Positive coping	0.88		0.55 - 1.41

Avoidant coping	1.42	**	1.22 - 1.65

Seeking social-support coping	0.73		0.54 - 1.00

Stigma scale	1.14	**	1.07 - 1.21

Disclosure (comparison group = none)			

Disclosed to some	2.97		0.56 - 15.83

Disclosed to all	4.33		0.92 - 20.37

Drink alcohol (yes/no)	0.69		0.30 - 1.55

Smoke tobacco (yes/no)	1.05		0.42 - 2.63

Smoke dagga (yes/no)	0.43		0.10 - 1.94

Sexual activity (comparison group = no sex)			

Always use a condom	1.52		0.91 - 2.54

Inconsistent condom use	1.51		0.58 - 3.93

Never use a condom	0.99		0.18 - 5.36

Financial status (comparison group = disability grant)			

Old-age pension	1.63		0.48 - 5.53

Child grant	2.12		0.45 - 9.92

Employed	0.75		0.37 - 1.52

Support within the household	0.64		0.32 - 1.27

Support outside the household	0.74		0.30 - 1.83

Other	1.30		0.57 - 2.95

Food supplements (comparison group = never)			

Previously	0.41		0.15 - 1.17

Currently	0.36		0.12 - 1.06

Time since first HIV + test	1.01	*	1.00 - 1.02

Side effects (comparison group = no side effects)			

Somewhat disruptive side effects	1.76		0.94 - 3.31

Very disruptive side effects	3.62	**	1.95 - 6.74

Health-related quality of life (EQ-VAS)	0.99		0.98 - 1.01

Sample size			499

Wald-statistic			106.8

P-value			< 0.001

R 2			0.22

% successfully predicted			78.56

Note: *p-value ≤ 0.05

**p-value ≤ 0.01

### Correlates of depression

The multivariate logistic regression analysis identified the following variables as correlates of depressive symptoms in people receiving ART (Table 2). Stigma was positively correlated with symptoms of depression (OR = 1.13, CI 1.06-1.20). The longer participants had known their HIV + status, the more symptoms of depression they were likely to have been experiencing (OR = 1.02, CI 1.01-1.03). Respondents who participated in a support group were less likely to have been depressed than those who had not belonged to a support group (OR = 0.21, CI 0.05-0.99). Finally, widowed study participants experienced fewer symptoms of depression than those who were single (OR = 0.30, CI 0.13-0.69).

**Table 2:** Multivariate logistic regression analysis of factors correlated with symptoms of depression in HIV + respondents on ARV treatment

	Odds Ratio	P-value	95% conf. Interval
District (comparison group = Lejweleputswa)			

Motheo	0.91		0.42 - 1.98

Fezile Dabi	0.84		0.37 - 1.89

Thabo Mofutsanyane	1.08		0.54 - 2.14

Xhariep	0.41		0.04 - 3.90

Female	0.93		0.50 - 1.72

Age (comparison group = under 30)			

30-34 years	1.21		0.54 - 2.70

35-39 years	1.72		0.76 - 3.87

40-44 years	1.68		0.73 - 3.89

45+ years	2.30		0.93 - 5.67

Marital status (comparison group = single)			

Cohabiting	0.75		0.38 - 1.48

Not cohabiting	0.64		0.26 - 1.59

Widowed	0.30	*	0.13 - 0.69

Separated or divorced	0.89		0.40 - 1.99

Education (comparison group = none)			

Primary	1.87		0.44 - 7.99

Some secondary	1.24		0.29 - 5.34

Matric and post-matric	1.11		0.23 - 5.30

Dwelling (comparison group = formal dwelling)			

Informal	0.82		0.39 - 1.74

Traditional	1.11		0.48 - 2.55

Other	0.92		0.39 - 2.15

Treatment buddy (yes/no)	1.20		0.88 - 1.64

Community worker (yes/no)	0.91		0.30 - 2.71

Support group (yes/no)	0.21	*	0.05 - 0.99

Positive coping	0.68		0.44 - 1.06

Avoidant coping	1.01		0.98 - 1.04

Seeking social-support group	1.05		0.75 - 1.49

Stigma scale	1.13	**	1.06 - 1.20

Disclosure (comparison group = none)			

Disclosed to some	0.43		0.09 - 2.08

Disclosed to all	1.18		0.29 - 4.90

Drink alcohol (yes/no)	0.83		0.38 - 1.79

Smoke tobacco (yes/no)	1.61		0.66 - 3.93

Smoke dagga (yes/no)	0.39		0.10 - 1.50

Sexual activity (comparison group = no sex)			

Always use a condom	0.90		0.53 - 1.51

Inconsistent condom use	1.12		0.45 - 2.77

Never use a condom	0.93		0.20 - 4.35

Financial status (comparison group = disability grant)			

Old-age pension	0.64		0.19 - 2.14

Child grant	1.47		0.31 - 7.01

Employed	0.74		0.37 - 1.49

Support within the household	0.73		0.36 - 1.49

Support outside the household	1.11		0.44 - 2.78

Other	0.68		0.26 - 1.78

Food supplements (comparison group = never)			

Previously	0.56		0.18 - 1.76

Currently	0.32		0.10 - 1.00

Time since first HIV + test	1.02	**	1.01 - 1.03

Side effects (comparison group = no side effects)			

Somewhat disruptive side effects	1.05		0.54 - 2.05

Very disruptive side effects	1.79		0.92 - 3.46

Health-related quality of life (EQ-VAS)	0.99		0.97 - 1.00

Sample size			499

Wald-statistic			82.26

P-value			< 0.001

R 2			0.17

% successfully predicted			78.96

Note: *p-value ≤ 0.05

**p-value ≤ 0.01

## Discussion

This study represents one of a few studies that systematically and simultaneously investigated correlates of symptoms of anxiety and depression in patients enrolled in a public sector ART programme in a resource-limited setting. Two risk factors, namely severe side effects and avoidant coping, were correlated with increased symptoms of anxiety, while a protective factor- participating in a support group- was associated with a decrease in symptoms of depression. Another risk factor, namely stigma, was associated with an increase in symptoms of both anxiety and depression.

Very disruptive ART side effects were correlated with more of the symptoms of anxiety amongst our study participants. Similarly, another study on ART in the Free State found that adverse side effects of treatment negatively affected the emotional well-being of the patients. The authors thus recommended that health care workers be vigilant regarding possible adverse side effects of treatment [42].

Our study did not find an association between coping strategies and depression [13,25,26,43]. However, unlike previous research, this study did investigate the association between coping strategies and anxiety. In this study, avoidant coping was correlated with a greater number of the symptoms of anxiety. It has been suggested that coping strategies of patients should be identified, and that patients using maladaptive strategies should receive therapeutic counseling [25].

Participating in a support group was associated with fewer symptoms of depression. This finding is in agreement with a study conducted in Thailand where low levels of social support were associated with increased levels of depression [19]. Social support is a protective coping mechanism that may assist HIV + people in solving problems and in expressing emotions, thereby reducing psychological distress [44-46].

In this study, perceived stigma was associated with symptoms both of anxiety and depression. This outcome confirms the findings from the literature that stigma is an important risk factor associated with depression [17,18,21,41]. The finding that stigma is associated with anxiety adds new evidence to the literature on the debilitating role that stigma may play in the lives of people receiving ART.

Whereas it is well established that widows and widowers face a greater risk of depression, especially in the immediate aftermath of bereavement [47], this study found widowhood to offer protection against symptoms of depression, which may be attributed to access to both material support and especially psychosocial support within their extended family and kinship networks [48-50].

The study has the following limitations; firstly, study participants were drawn only from those HIV + individuals who had gained access to the public sector antiretroviral treatment programme and had successfully completed drug-readiness training. Patients suffering from anxiety and depression may be less likely to seek care, complete drug readiness, and initiate treatment. They are thus under-represented in this study. Secondly, because the empirical analysis was based on self-reported information, the measures may not be completely accurate. Particularly problematic variables include self-reported side effects and sexual behaviour. Thirdly, this was a cross-sectional study, so that all the data were measured at the same time. Consequently, causality between symptoms of anxiety and depression and their correlates could not be firmly established. Fourthly, although according to some authors "the HADS appears to represent the best currently available self-report to reliably and validly assess anxiety and depression in HIV infected patients" [34], it has not yet been validated among HIV + patients initiating ART in South Africa. In the fifth instance, the Cronbach's alpha for the two subscales is below the threshold of 0.70. The scale may thus not be all that reliable [38]. For the purposes of this study, the instrument was translated into SeSotho. Further research is consequently required so as to ensure the reliability and validity of the translated version. Finally, by using a low cut-off of 8+ the study may be missing important correlates of mild (8-10), moderate (11-15) and particularly severe (16-21) symptoms of anxiety and depression [51]. The small number of severe cases observed in this study [anxiety n = 7, depression n = 4] precluded meaningful statistical analysis. In larger samples, multivariate multinomial logistic regression offers a method by which jointly to determine factors correlated with each of the four classes of symptoms of anxiety and depression.

## Conclusions

This study sheds some light on the nature of symptoms of anxiety and depression in patients receiving ART in a resource-limited setting. The prevalence of symptoms of anxiety was 30.6% and that of depression was 25.4%. Severe side effects of ART, avoidant coping strategies, participating in a support group and stigma are correlates of anxiety and/or depression.

This study has both theoretical and practical implications.

From a theoretical point of view, the impact of ART side effects, coping strategies, support groups and stigmatisation on the mental health of HIV/AIDS patients draws attention to the inter-linked nature of treatment aspects, social support and mental health in achieving durable ART success. However, further research is required so as fully to disentangle the complex interrelationships between these social, mental and physical aspects of public-sector ART in high HIV-prevalence resource-limited settings.

From the perspectives of practical policy and management, the study findings provide valuable insights into psychosocial aspects of the Free State public-sector ART programme. Combined with the literature on the intricate link between mental health problems and treatment outcomes our results emphasise firstly, the necessity that resources be allocated for screening and treating mental-health problems, and, secondly, that interventions are required that will encourage support-group participation, address ART side effects, reduce maladaptive coping styles and minimise stigma associated with symptoms of anxiety and/or depression.

## Appendix: Details of measures

Data on demographic, socio-economic, behavioural, psychosocial and health variables were collected. *Demographic variables *included 'district of residence', 'gender', 'age' and 'marital status'. The variable *marital status *comprised 'single', 'cohabiting', 'not-cohabiting', 'widowed', and 'separated or divorced'. 'Single' refers to participants without any type of partner, 'cohabiting' refers to participants who lived with a partner irrespective of whether or not they were married, and 'not-cohabiting' refers to participants whose partners (married or not) lived elsewhere at the time of the interview.

*Socio-economic *variables included 'education level', 'dwelling type', 'financial status', and 'food supplements'. *Education level *comprised four categories: 'none', 'primary', 'some secondary', 'matric' (Grade 12) and 'post-matric'. *Dwelling type *comprised four categories: 'formal', 'informal', 'traditional', and, 'other'. *Other *included 'a flat or apartment in a block of flats', 'a dwelling in a backyard' and 'a hostel'. *Financial status *refers to how participants usually support themselves. The seven categories were: 'disability grant', 'old-age pension', 'child-support grant', 'employed', 'support within the household', 'support outside the household', and,'other'. The variable *food supplements *referred to the respondents' having received food supplements as part of the government's ARV treatment programme. The categories were 'never', 'previously' or 'currently'.

*Behavioural variables *included 'use of alcohol', 'use of tobacco', 'use of dagga', and 'engagement in sexual activity'. 'Use of alcohol', 'use of tobacco', and 'use of dagga' were dichotomous variables, referring only to whether or not a person engaged in the activity rather than establishing the extent to which the person was doing so. *Sexual activity *referred to whether or not participants had had sexual intercourse in the six months prior to the study, and if so, whether they had used a condom. The four categories for this variable were: 'no sex', 'always used condom', 'inconsistent condom use' and 'never used a condom'.

*Psychosocial factors *included 'stigma', 'coping', and 'psychosocial support'. The *stigma scale *was generated from eight items in the questionnaire, asking respondents about the extent to which they agreed or disagreed with perceptions that they or others had regarding HIV and AIDS. The possible responses were: 'strongly disagree', 'disagree', 'agree' or 'strongly agree' [52]. The scale reported an alpha of 0.70 [31]. Three *coping *behaviours were measured: 'positive', 'avoidant' and 'seeking social support coping styles'. Respondents were asked to describe how they were currently (at the time of the study) dealing with living with HIV and AIDS by answering 'yes' or 'no' to sixteen statements. The statements were taken from a study conducted in the United States between 1988 and 1989. In that study, a factor analysis of the sixteen coping behaviours of 1031 people with AIDS revealed 'positive', 'avoidant' and 'seeking social support' to be three main coping mechanisms of people with AIDS [26]. *Psychosocial support *included four questions concerning 'a treatment buddy', 'community worker', 'support group' and 'disclosure', the first three of which were dichotomous variables. 'Treatment buddy' refers to whether the respondent at the time of the present study had a treatment buddy; 'community worker' denotes whether the participant at the time had a community worker visiting the participant at home; and 'support group' refers to whether the participant at the time of the study belonged to a support group. 'Disclosure' refers to the extent to which participants had disclosed to the three most important people in their lives, with response categories of 'none', 'some' and 'all'.

*Health variables *included 'health-related quality of life' (EQ-VAS), 'time since first HIV + test', and 'side effects'. 'EQ-VAS' is a visual analogue scale that measures a participant's health "today" and ranges from 0% (worst health) to 100% (best health). 'Time since first HIV *+ *test' refers to the number of months the study participant had known their HIV + status. 'Side effects' refer to whether the participant experienced side effects from ART, and, if so, how disruptive these side effects were: 'no side effects', 'somewhat disruptive' or 'very disruptive'.

## Competing interests

The authors declare that they have no competing interests.

## Authors' contributions

MP: Analysis of data, interpretation of results and original manuscript. EW: Revision of manuscript for important intellectual content. FLRB: Revision of manuscript for important intellectual content. All of the above authors read and approved the final manuscript.

## Pre-publication history

The pre-publication history for this paper can be accessed here:

http://www.biomedcentral.com/1471-2458/12/244/prepub
